# SSEA-4 positive dental pulp stem cells from deciduous teeth and their induction to neural precursor cells

**DOI:** 10.1186/s13005-022-00313-6

**Published:** 2022-03-02

**Authors:** Ada Pricila Lopez-Lozano, Katiushka Arevalo-Niño, Yolanda Gutierrez-Puente, Jose Luis Montiel-Hernandez, Victor Hugo Urrutia-Baca, Casiano Del Angel-Mosqueda, Myriam Angelica De la Garza-Ramos

**Affiliations:** 1 Facultad de Ciencias Biológicas, Instituto de Biotecnología, Universidad Autónoma de Nuevo León, Nuevo Leon San Nicolas de los Garza, Mexico; 2 Unidad de Odontología Integral y Especialidades, Centro de Investigación y Desarrollo en Ciencias de la Salud, Universidad Autonoma de Nuevo Leon, Nuevo Leon Monterrey, Mexico; 3 Facultad De Farmacia, Coordinacion De Posgrado, Universidad Autonoma del Estado de Morelos, Morelos Cuernavaca, Mexico; 4 Facultad de Odontología, Universidad Autonoma de Nuevo Leon, Nuevo Leon Monterrey, Mexico; 5 Facultad de Odontología/CIDICS, Universidad Autonoma de Nuevo Leon Monterrey, San Nicolás de los Garza, Mexico

**Keywords:** Dental pulp, Deciduous teeth, Neuroesphere assay, Neural differentiation, Neural precursor cells

## Abstract

**Background:**

Stage-specific embryonic antigen-4 (SSEA-4) is a marker for the identification of multipotent embryonic cells. It is also positive in neuroepithelial cells, precursor neural cells (NPC), and human dental pulp cells. The aim of this study was to evaluate the potential morphodifferentiation and histodifferentiation to NPC of SSEA-4 positive stem cells from human exfoliated deciduous teeth (SHED).

**Methods:**

A SHED population in culture, positive to SSEA-4, was obtained by magnetic cell separation. The cells were characterized by immunohistochemistry and flow cytometry. Subsequently, a neurosphere assay was performed in a medium supplemented with basic fibroblast growth factor (bFGF) and epidermal growth factor (EGF); afterward, cells were neurodifferenciated with a neurobasal medium. Finally, indirect immunohistochemistry was performed to identify neuronal markers.

**Results:**

The morphological and histological changes in the SSEA-4 positive SHEDs were observed after induction with epidermal and fibroblast growth factors in neurobasal culture medium. At the end of induction, the markers Nestin, TuJ-1, and GFAP were identified.

**Conclusions:**

The findings show that SSEA-4 positive SHEDs have a behavior similar to neuronal precursor cells. Our findings indicate that the dental pulp of deciduous teeth is a promising source for regeneration therapies associated with neurodegenerative diseases or peripheral nerve alterations.

## Introduction

Neural precursor cells (NPC) in the nervous system (SN) can differentiate to neurons, astrocytes, and oligodendrocytes. In contrast to neural stem cells (NSC), NPC have limited self-renewal [1]. It was previously thought that this population of cells resided only in the human brain in development. Later, the presence of NPC was reported in the adult brain [1, 2].

Degenerative diseases, traumatic events, and injuries that occur during or after medical procedures generate neuronal alterations, inflammation, cell death, and cytoarchitecture malformations in the central nervous system (CNS) and peripheral nervous system (PNS). Conventional medical therapies provide limited efficacy in the recovery of function after nerve damage. Because of nervous system maturation, and despite the presence of NPC, the ability to generate new neurons and glial cells is reduced and limited with increasing age [3].

Treatments based on mesenchymal stem cells (MSC) induce neuronal regeneration. These cells are located in bone marrow, adipose tissue, the umbilical cord, and the orofacial region [4]. MSC have been shown to have multiple neurotrophic and anti-inflammatory factors that favor nerve repair and serve as potentials candidates for cell therapy for CNS and SNP alterations.

The niches of MSC in the orofacial region are the dental pulp tissue of deciduous and permanent teeth, the dental follicle, the periodontal ligament, the oral mucosa, and the bichat fat pad [5, 6].

In pulp tissue, human dental pulp stem cells (DPSC) are MSC isolated from permanent teeth; stem cells from human exfoliated deciduous teeth (SHED) are in temporary teeth. Both cell populations come from cells in the groove of the neural plate in development; these are highly clonogenic and have multilineage differentiation, contributing to the equilibrium and specific conditions for tissue repair [7].

One of the relevant differences between DPSCs and SHED is that both cell populations can be obtained from individuals from different age groups, making them capable of expressing different gene types [8]. In particular, SHED, because of their origin and initial formation, can express embryo-stage genes such as stage-specific embryonic antigen-4 (SSEA-4), an antigen used to identify NPC [9, 10].

The objective of this study was to analyze the morphodifferentiation and histodifferentiation ability of a neural lineage of deciduous teeth mesenchymal cells obtained by magnetic separation with SSEA-4 antibody. We hypothesized that a cell population similar to NPC exists in human exfoliated deciduous teeth in the dental pulp.

## Materials and methods

### Pulp tissue collection

Pulp tissue was obtained from 40 deciduous teeth from children between 6 and 10 years of age. The included children were of both sexes with no clinical-pathological data and with healthy teeth in a state of exfoliation, candidates for extraction by therapeutic enucleation (germectomy), and orthodontic or orthopedic treatment. Children with any disease, less than 6 or greater than 10 years of age, with healthy teeth but no indication for extraction or with teeth with some pathology were excluded. The parents or guardians had to accept the participation of their child and sign a written informed consent. The Ethics Committee of the School of Dentistry of the Autonomous University of Nuevo Leon evaluated and approved the protocol.

### Dental extraction

The study was performed with a total of 40 teeth with one exclusive assay for each patient sample. The indications given to each participant (patient or parent-guardian) were to perform dental prophylaxis at the dental office one week before obtaining the sample, tooth brushing daily, 3 times a day, and a diet low in carbohydrates. On the day of sample collection, second prophylaxis and direct cleansing of the tooth surface for 5 min with gauze soaked in 2% chlorhexidine (Consepsis-Ultradent) were performed. The dental samples obtained were transported to the laboratory in 50 ml Falcon tubes (Corning) with 1X phosphate-buffered saline (PBS), 100 µg/mL streptomycin, 100 U/mL penicillin, and 0.25 mg/mL amphotericin B (Sigma).

### Dental pulp extraction

Odontosection of the extracted dental samples was performed at the level of the cementoenamel junction (limits of the crown and the dental root) with a medium diamond grain disk (NTI), mounted on a low-speed electric micromotor (Dremel) with constant irrigation with 0.9% sterile saline solution (PISA). In samples with an open radicular apex, this was used as an access to remove the pulp tissue with no.20 Triple Flex endodontic extractors and files (Kerr).

Subsequently, the extracted tissue pulp was placed in 2 mL Eppendorf tubes with 1 mL 1X PBS, 100 µg/mL streptomycin, 100 U/ml penicillin, and 0.25 mg/mL amphotericin B (Sigma) and preserved in refrigeration at 4 °C without exceeding 48 h after the extraction.

### Tissue dissociation

The tissue pulp was dissociated in a GentleMACS™ dissociator in a C tube (Miltenyi Biotec) with 4.7 mL 1X PBS. After dissociation, the sample was centrifuged at 300x g for 10 min.

After obtaining the cell pellet, 3 mg/mL of collagenase type I (Sigma) and 4 mg/ml of dispase (Sigma) were added. The suspension was then incubated for 30 min at 37 °C. After incubation, the suspension was centrifuged again at 300x g for 10 min. Subsequently, the supernatant was removed and the enzymes inactivated by washing with 5 mL DMEM F12 medium (Gibco) and 10% fetal bovine serum (FBS) (Gibco). The suspension was filtered with an 80 μm membrane (Millipore); finally, it was resuspended in 1 mL culture medium.

### Cell count

A hemacytometer or Neubauer chamber (Marienfeld) was used to determine the number of cells and their viability. An aliquot of 10 µl of the cell suspension and 10 µl of trypan blue 0.05% (Sigma) was obtained with a micropipette (Eppendorf) before homogenization; the 20 µl were mixed in a 2 ml Eppendorf tube and placed in Neubauer chamber for cell counting.

### Cell culture

Dulbecco’s Modified Eagle’s Medium/Nutrient Mixture F12 Ham (DMEM-F12), 10% FBS (Gibco), 100 mg/mL streptomycin, 100 U/ml penicillin, and 0.25 µg/ml amphotericin B (Sigma) were used for the primary cell culture (PCC). The counted cell suspension was resuspended in 5 mL of culture medium and placed in 25 cm^2^ Falcon vials (Corning) for cell culture.

The PCC was kept in a chamber with 5% CO_2_ at 37 °C (Thermo Scientific). After 72 h, the non-adherent cells were removed; the culture medium was changed every 3 days. Subsequently, the culture medium was removed, and the cells adhered to the culture flask were washed three times with 1X PBS. Then, cell trypsinization was performed with 0.25% trypsin and 1 mM EDTA (Fisher Scientific). Trypsin/EDTA was added, and the solution was incubated for 5 min at 37 °C in a chamber with 5% CO_2_. After incubation, the enzyme was inactivated with the culture medium in a proportion equal to the volume of the content in the flask; it was then centrifuged at 300x g for 10 min. The cells were resuspended in the culture medium and centrifuged; later, they were resuspended in 1X PBS with 1% bovine serum albumin (BSA) (Sigma-Aldrich). Magnetic microbeads of 50 nm conjugated with SSEA-4 antibody (MACS/Miltenyi Biotec) were incubated with the cell suspension for 15 min at 4 °C to achieve stage 3 cell growth and expansion. The separation of mesenchymal stem cells derived from the dental pulp was performed using a MACS magnetic separator system (Miltenyi Biotec). Finally, the content of the 15 mL tubes (Corning) was collected to recover the SSEA-4 antibody-positive fractions.

### Flow cytometry staining

Staining for flow cytometry was performed with the antibodies CD45 FITC, CD14 PE (present in hematopoietic stem cells), CD13 PE, CD44 FITC, and CD105 PE (present in mesenchymal stem cells) to identify and study the cell population in the SSEA-4 positive fraction (Table 1).

**Table 1:** Monoclonal antibodies used for identification of mesenchymal cell membrane markers

Origin	Antigen	Manufacturer
Mouse	CD44 FITC	Miltenyi Biotec
Mouse	CD71 FITC	Miltenyi Biotec
Mouse	CD90 FITC	Miltenyi Biotec
Mouse	CD146 FITC	Miltenyi Biotec
Mouse	CD13 PE	Miltenyi Biotec
Mouse	CD105 PE	Miltenyi Biotec

*FITC* fluorescein-5-isothiocyanate, *PE* phycoerythrin

### Direct immunocytochemistry

Immunohistochemistry was performed with primary antibodies to identify mesenchymal cell markers. These cells were seeded in 16-well chamber slides for cell culture (Nunc™ Lab-Tek). The culture medium was removed when the cells reached a confluence of 80% inside each well. The cells were washed with 1X PBS and placed in methanol at − 2 °C for 10 min to fix the cells. Afterward, they were incubated at environmental temperature with 1X PBS and 2% BSA for 30 min. After 30 min, the antibodies for identification were added, and the cells were incubated for 3 h at environmental temperature and in darkness.

At the end of the incubation time, the supernatant was removed, and 3 washes were performed with 1X PBS. To identify nucleic acids, 1:1000 DAPI (Sigma-Aldrich) was added; the cells were incubated for 15 min in darkness, and one wash was performed with 1X PBS.

After staining, the structures in the slide chambers were removed, 10 µl of mounting solution (Dabco) was added, and a coverslip was placed on each field for analysis. The coverslips were sealed to the slide with enamel, and the slides were analyzed with an inverted fluorescence microscope (Zeiss Z1, Carl Zeiss, USA).

### Neuronal differentiation culture

The neuronal differentiation protocol was divided into two stages with different culture media: Neuronal A medium (NA) (neurosphere assay) pre-induction to maintain multipotentiality, growth, and division of neurospheres from SHED; and neuronal B medium (NB), directed at cell differentiation.

#### Neuronal A medium (neurosphere assay)

The components of the pre-induction medium were DMEM-F12 (Gibco), 50 µl/mL Supplement (Gibco), 2% FBS (Gibco), 3 µl/mL L-glutamine (Gibco), 20 ng/mL epidermal growth factor (EGF), 20 ng/mL fibroblast growth factor (FGF), 100 µl/mL streptomycin, 100 U/mL penicillin, and 0.25 mg/mL amphotericin B (Sigma-Aldrich). The cells previously separated by magnetic microbeads conjugated with SSEA-4 antibody were placed in a 25 cm^2^ culture vial (Corning) for expansion. After performing the second cell passage, the cells were trypsinized and reseeded in 5 mL NA to await neurosphere formation in the pre-induction stage for 7 days; half of the medium was changed every 3 days.

#### Neural medium B

The components of the cell differentiation medium were half Neurobasal (Gibco), 70 µl/mL of supplement B27, 3 µl/mL L-glutamine (Gibco), 500 nM retinoic acid (Sigma Aldrich), 100 µg/mL streptomycin, 100 U/mL penicillin, and 0.25 mg/mL amphotericin B (Sigma-Aldrich). The cells were kept in NB for 7 days; half of the medium was changed every 3days.

The cells were seeded on slides with two-well chambers for cell culture (Nunc™ LabTek). For cell adhesion, the surface of the slide was prepared with 0.1 mg/mL of Poly-L-Lysine (Sigma-Aldrich). A total of 60 µl was added to each well and kept in incubation for 12 h at 37 °C. Later, the supernatant was removed, and each well was washed with 500 mL milli-Q (mQ) double distilled water. The cells were seeded in 400 µl NB and kept in culture for 7 days; half of the medium was changed every 3days.

### Indirect immunohistochemistry

After seven days in NB, indirect immunohistochemistry was performed to identify neuronal markers using secondary antibodies for Nestin, B-III Tubulin (TuJ-1), and glial fibrillary acidic protein (GFAP).

After the culture plates were observed in a phase-contrast microscope, the NB medium was removed, and the cells were washed with 400 µl 1X PBS. The cells were fixed with methanol at − 2 °C for 10 min; the supernatant was removed, and 2 washes were performed with 1X PBS.

The cell membrane was permeabilized with 0.5% Triton X-100, and the cells were incubated for 5 min. After incubation, 3 washes of 5 min with 1X PBS were performed; then, a blocking solution was added (10% fetal goat serum [FGS] in 1X PBS). The cells were then incubated for one hour at environmental temperature to avoid unspecific reactions.

For antibody dilution-titration, 1% FGS with 1X PBS was prepared, adding the primary antibodies (mouse-anti-human), anti-Nestin, anti-B-III tubulin, and anti-GFAP. The solution was incubated for 12 h (during the night) at 4 °C. The supernatant was removed the next day, and 3 washes of 5 min with 1X PBS were performed. Subsequently, the secondary antibodies anti-Nestin, anti-B-III tubulin, and anti-GFAP (antibodies conjugated with Alexa Fluor 568) (rabbit-anti-mouse) were added. This solution was incubated for one hour at environmental temperature and in the dark. After incubation, 3 washes of 5 min with 1X PBS were performed.

For cell nucleus staining, 1:1000 DAPI (Sigma-Aldrich) was added, and the cells were left to incubate for 10 min in the dark. After removing DAPI, one wash with 1X PBS was performed.

After staining with primary and secondary antibodies, the structure of the chambers of the slides was removed, 10 µl of mounting solution (Dabco) was added, and a coverslip was placed in each field for analysis. The mounting solution drained through the coverslip for 24 h to remove the excess; eventually, the coverslip was sealed with enamel, and the analysis was performed with an inverted fluorescence microscope (Zeiss Z1, Carl Zeiss, USA).

## Results

### Obtention of biological samples from teeth

The teeth were from dental enucleations (permanent premolar germ extractions before normal eruption), teeth exfoliation, and serially extracted teeth. The majority were male patients; the age range was 8 to 9 years. The number of cells obtained per sample was variable, but a mean of 50,000 viable cells was considered.

**Fig. 1:**
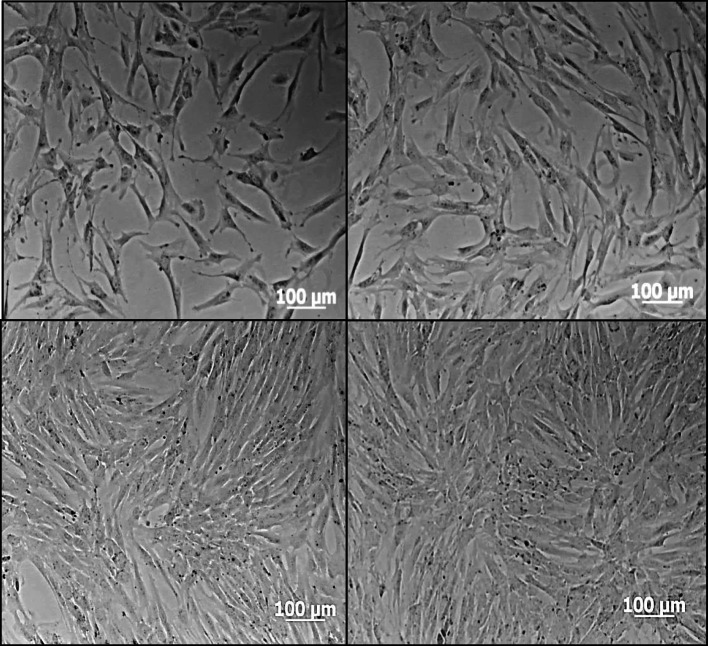
Microphotographs obtained with a phase contrast microscope. Initial culture - Step 0 (PO) dental pulp cells from deciduous teeth in growth medium. **A** 50% confluence, (**B**) 60% confluence, (**C**) 70% confluence, (**D**) 80% confluence

The replication time of the human dental pulp cells was 21.89 h. The cells adhering to the culture vial had a heterogeneous appearance in shape and size, a fusiform morphology with some relatively short and wide or rather long, thin, and very branched cytoplasmic extensions, oval nuclei, and scarce cytoplasm. They showed fast growth, reaching 80% confluence by day 10 and 100% by day 14. Three cell passages were performed, and in each, the expansion rate increased (Fig. 1).

### Isolation and characterization of SHED

**Fig. 2:**
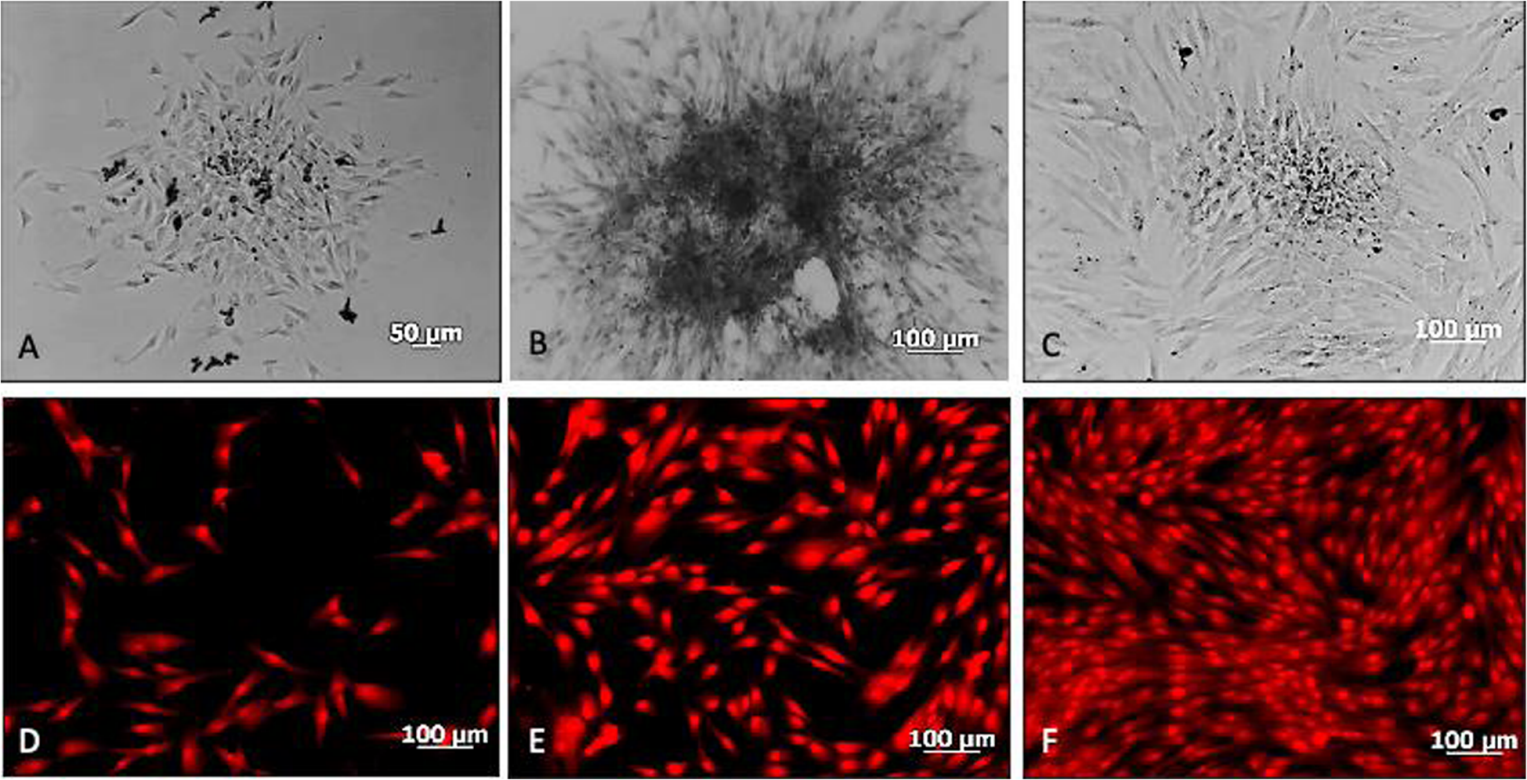
Mesenchymal cell culture from deciduous dental pulp, positive for the SSEA-4 antibody. **A, B, C**: Microphotographs obtained with a phase contrast microscope on days 3, 5, and 7 of culture in growth medium showing the cell organization and growth in colony forming units (CFUs). **D, E, F**, Microphotographs obtained with an inverted fluorescence microscope where an autofluorescence assay with formalin is shown on days 3, 5, and 7, of culture in growth that shows the growth pattern in CFUs and homogeneous cell morphology

The mesenchymal cells positive to magnetic microbead separation conjugated with the SSEA-4 antibody were adherent to the culture vial, had a homogeneous morphology in size, and a spindle shape, an oval nucleus, scarce cytoplasm, a growth characteristic in colonies or colony-forming units (CFUs) (approximately 100 cells per CFU) and a high expansion rate, similarly to the not purified culture (Fig. 2).

**Fig. 3:**
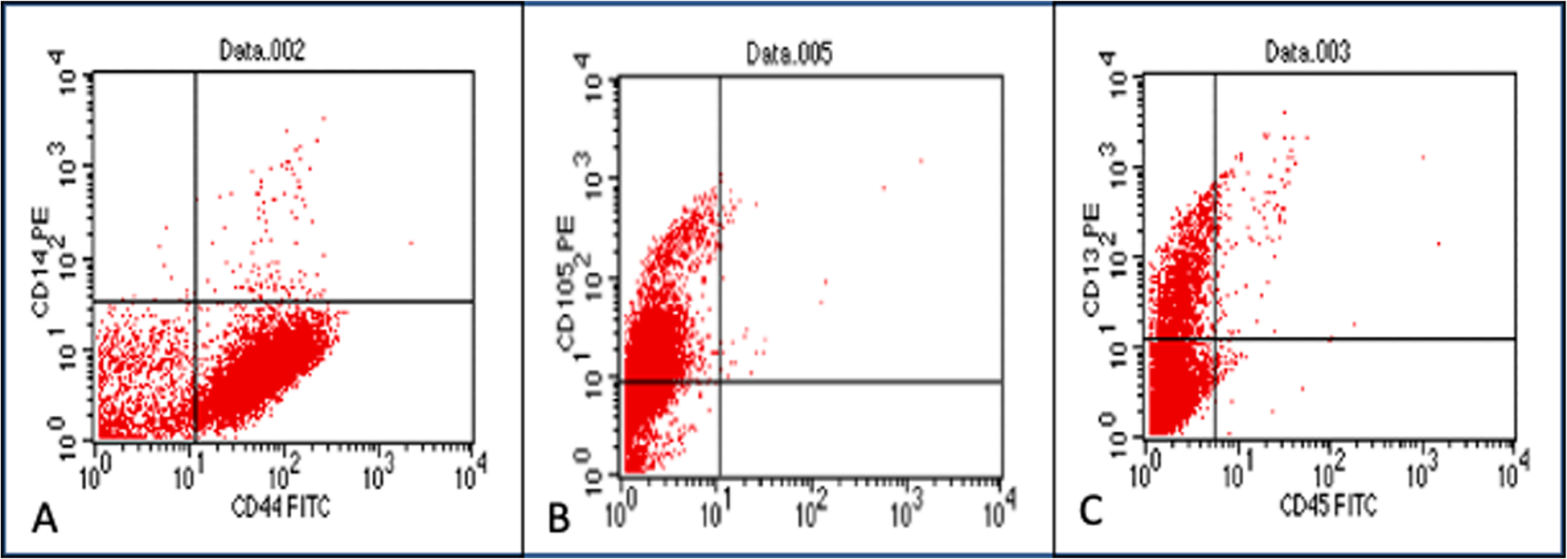
Dot plots obtained with a flow cytometer. **A** CD14 (-), CD44 (+) homogeneous cell population; (**B**) CD105 (+) homogeneous cell population; (**C**) CD13 (+), CD45 (-), homogeneous cell population

Flow cytometry identified the immunophenotype of the cell population isolated with magnetic microbeads and SSEA-4 antibody. The cell population characterization by immunocytochemistry was positive for the mesenchymal cell membrane markers CD44, CD71, C13, and CD105, and negative for the hematopoietic antibodies CD45 FITC, CD14 PE. Characterization of the cell population with direct immunocytochemistry was positive for mesenchymal cell membrane markers (Fig. 3; Table 2).

**Table 2:** Stem cells from human exfoliated deciduous teeth (SHED) immunophenotype

Membrane marker	Expression	Immunophenotype
CD44 FITC	(+)	MSC
CD105 PE	(+)	MSC
CD13 PE	(+)	MSC
CD14 PE	(−)	HSC
CD45 FITC	(−)	HSC

*FITC* fluorescein-5-isothiocyanate, *PE* phycoerythrin, *MSC* mesenchymal stem cells, *HSC* hematopoietic stem cells

### Neural differentiation

**Fig. 4:**
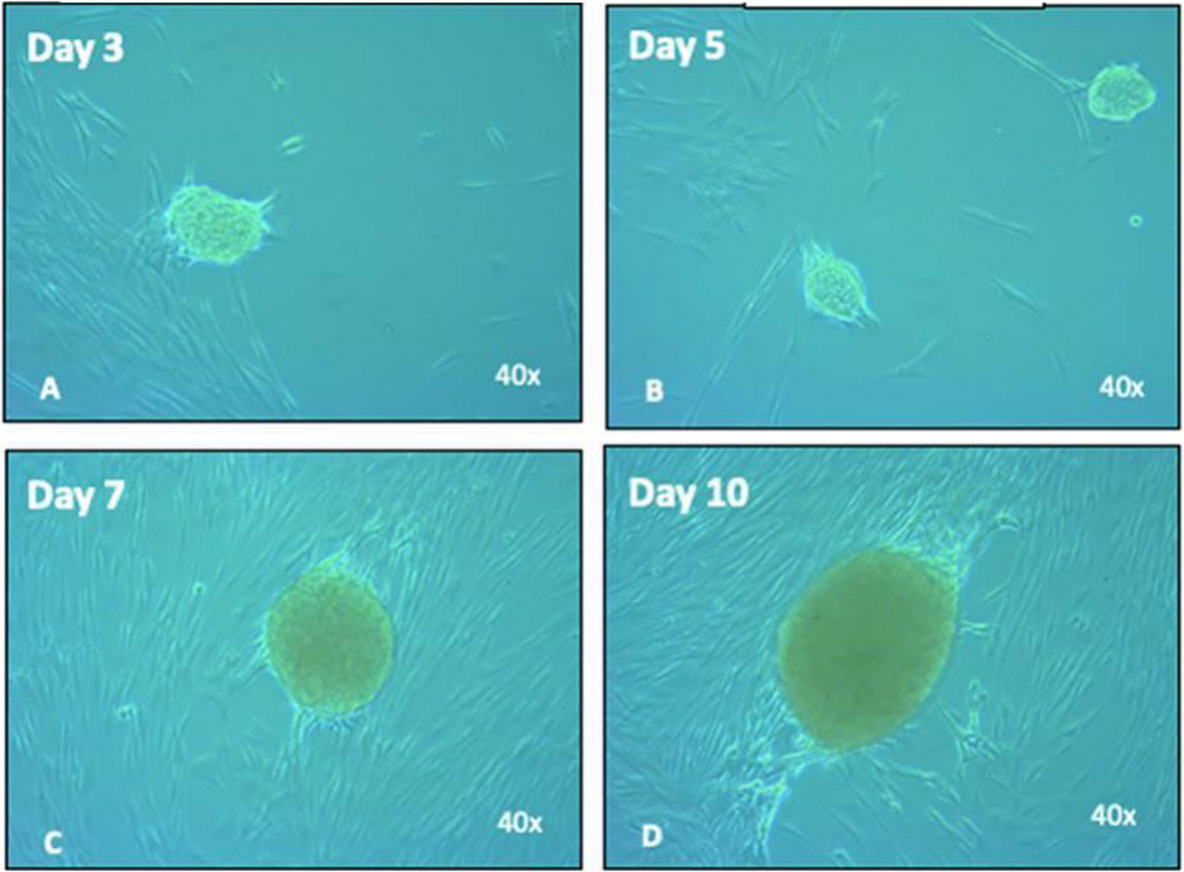
Neurosphere development in neuronal medium A. **A** Neurosphere formation was observed from the third day; appearance of a structure with a condensed center, a perimeter halo, and development in suspension (non-adherent). **B** On day 5, an increase in the number of neurospheres was observed. **C** From the seventh day, the beginning an increase in the size of the neurospheres was observed. **D** Cells in continuous division increasing the dimensions of the spheroid structure in suspension

The cells in neuronal A medium initially presented as a monolayer adherent to the culture vial with a fibroblastic morphology organized in CFUs. In the next 48 to72 hours, an increase in the cell density of each CFU was observed. The cells gradually changed their morphologies, longitudinally compacting the colony. By the third day, an accumulation of cells losing adhesion to the surface of the culture vial was observed, forming bodies with a spheroid shape and a mesenchymal cell structure. The spheroid bodies or neurospheres kept developing and growing in the pre-induction medium until the tenth day (Fig. 4).

**Fig. 5:**
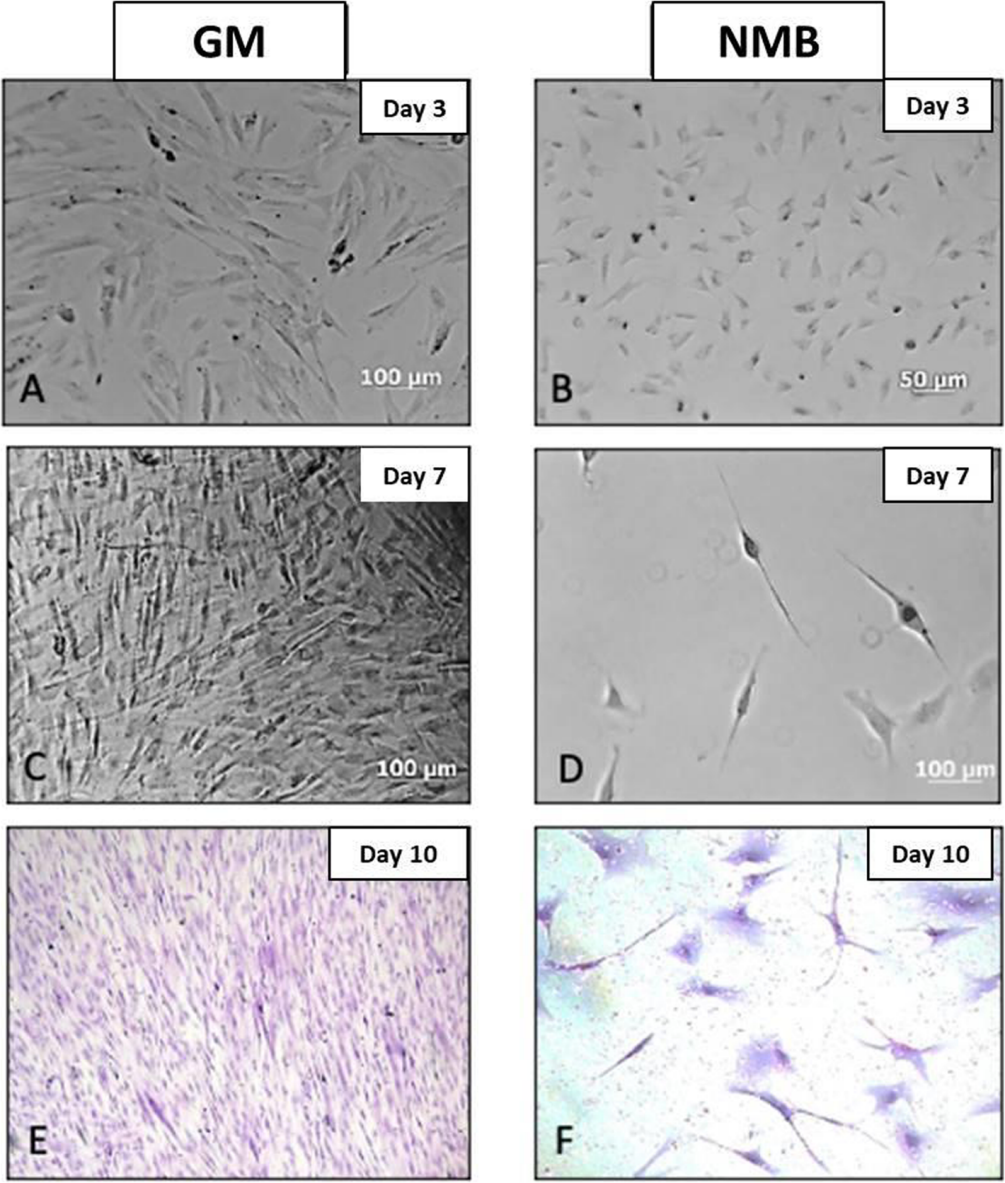
Neuronal differentiation culture. **A** Mesenchymal cells in growth medium (GM) at day 3. **B** Mesenchymal cells from neurospheres in neuronal medium B (NMB) at day 3. **C** Proliferating mesenchymal cells in GM day 7. **D** Neuronal cells in the process of morphological differentiation day 7. **E** Mesenchymal cells with 80% confluence at day 10. **F** Neuronal cells differentiated at day 10 in NMB

The cells derived from the neurospheres, seeded on a surface treated with Poly-L-lysine and in neuronal B medium, presented radical morphological modifications, starting with a mean size of 20–50 μm (Fig. 5). In differentiation culture induced with retinoic acid, they increased in length by more than 50% by issuing multiple extensions with condensed well-defined and apparent nuclei, a cell body or soma showing scarce cytoplasm and granules in its interior. Cell density considerably decreased by inducing a neuronal differentiation of more than 70%.

### Identification of neuronal markers

**Fig. 6:**
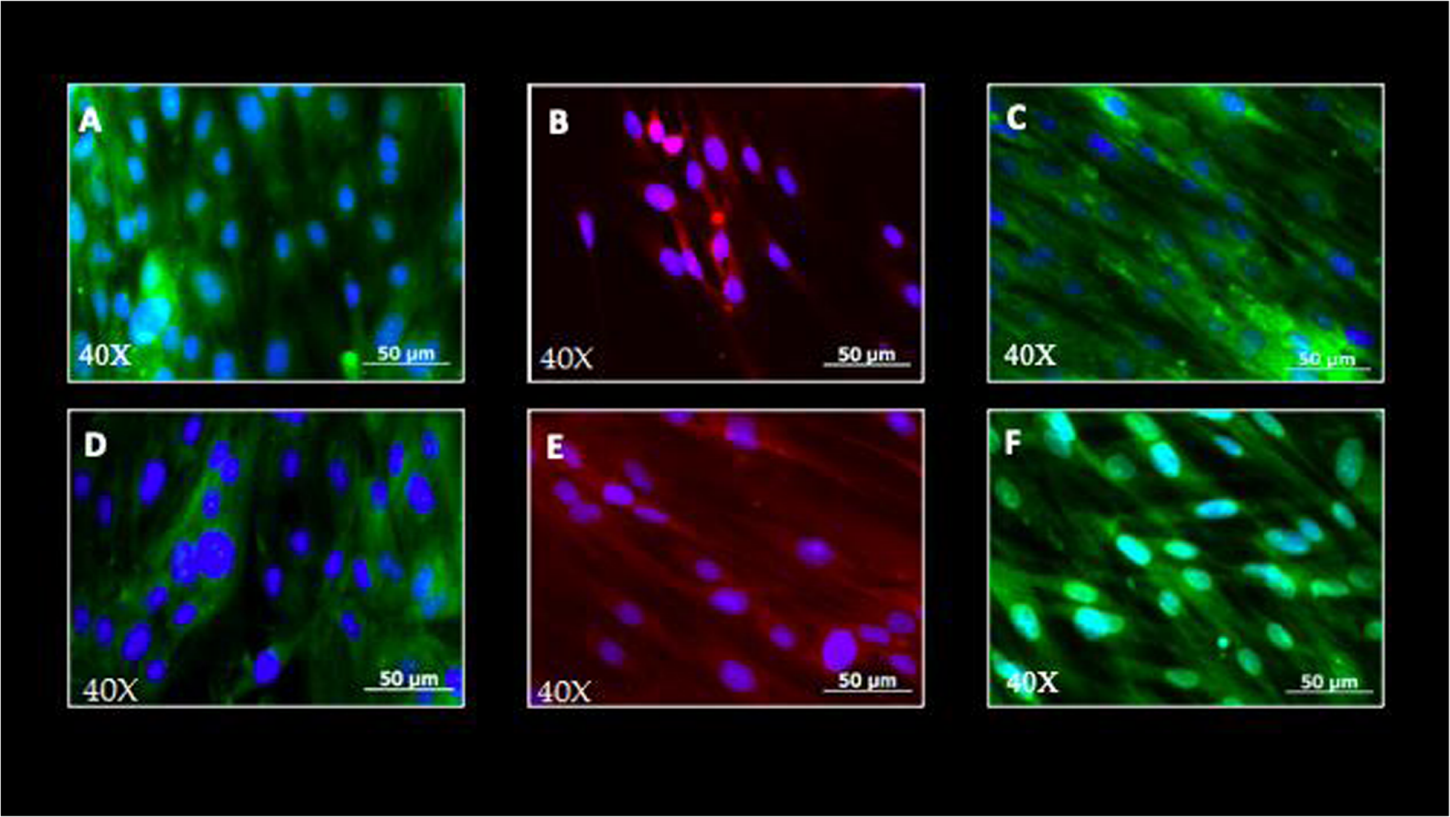
Immunocytochemistry for identification of mesenchymal cell membrane markers. **A** CD44 FITC +, (**B**) CD13 PE +, (**C**) CD71 FITC +, (**D**) CD90 FITC +, (**E**) CD105 PE +, (**F**) CD146 FITC. Analysis obtained with an inverted fluorescence microscope

**Fig. 7:**
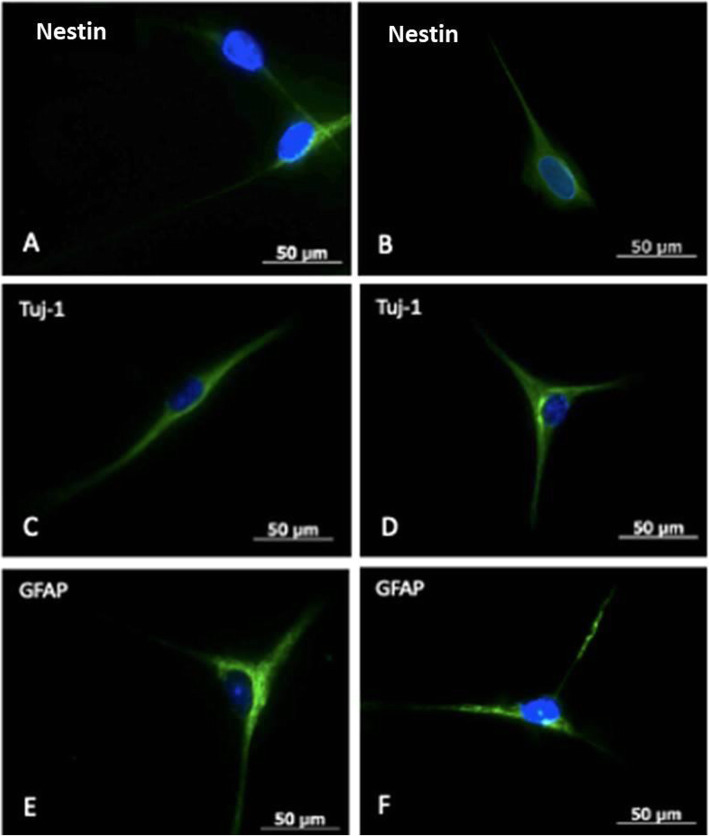
Immunocytochemistry for identification of neuronal cell membrane markers. **A** and **B** Nestin marker positive neuronal cells. **C** and **D** Tuj-1 marker positive neuronal cells. **E** and **F** Glial fibrillar acid protein (GFAP) marker positive neuronal cells. Analysis obtained with an inverted fluorescence microscope

The immunocytochemistry assay after induction with NB for 10 days was positive for the marker nestin, present in neuroepithelium or precursor neural cells; B-III Tubulin (TuJ-1), positive for neuronal activity and related with neurogenesis and axonal growth; and glial fibrillar acid protein (GFAP), present in the intermediate filaments of the cytoskeleton, mainly in glial cells (Figs. 6 and 7).

## Discussion

The dental pulp is a specialized tissue generated from ectomesenchyme formed by the ectoderm of neuroepithelial and mesoderm cells rich in different cell populations [11, 12].

In this study, samples were collected from children between 6 and 9 years of age. The samples were teeth from natural exfoliation or indicated for removal for therapeutic reasons; enucleated premolars from 9-year-old children were also included. This study is the first report of research with this type of sample. Others have been documented with dental germs from third molars of adult patients [13].

This study is the first time that magnetic separation with the SSEA-4 antibody in a population of mesenchymal stem cells from human exfoliated deciduous teeth (SHED) is reported. Another study has identified dental pulp stem cells (but not the purification of a cell population with this antibody) by flow cytometry [11]. On the other hand, regarding this cell marker in the embryo stage, it has also been identified as a cell marker in states of neuronal differentiation [14, 15].

SSEA-4 is a specific antigen that can isolate and purify stem cells from human exfoliated deciduous teeth. The obtention of mesenchymal cells by magnetic separation with the SSEA-4 antibody is relevant because this antigen is expressed in embryo pre-implantation stages, embryogenesis, and subsequently, in undifferentiated mesenchymal cells [16]. We can deduce that a cell population that expresses SSEA-4 exists in the dental pulp of deciduous teeth; in the same way, neuroepithelial and neuronal progenitor cells express it during differentiation. These findings allow us to compare the potential of our cell population with naïve cells that present pluripotency [10, 17, 18].

The SHED obtained by magnetic separation were characterized and identified as a cell population purified from mesenchymal cells, confirmed by immunocytochemistry and flow cytometry, and obtaining positive results for antibodies that correspond to mesenchymal cells and negative for hematopoietic cells antibodies as described by Bianco et al. [19] for the identification of mesenchymal cells. In culture, these cells were dependent on surface adhesion with a spindle shape and organized in highly clonogenic colonies [20, 21].

The results of this research provide evidence that supplementation of the SHED culture with the growth factors EGF, FGF, and a low concentration of FBS, leads to the rapid formation of neurospheres from day 3, compared with other studies that report their formation after 6 days and with treatment carried out with DPSC [22, 23].

It is important to point out that this work was performed with dental pulp from deciduous teeth because children in the early stages of human growth and development have cells with a greater potential for proliferation and differentiation than cells from adults.

The process of neurogenesis in the postnatal period requires multiple steps, such as proliferation, differentiation, migration, cell growth, and synaptic integration. Each of these stages expresses a different marker or antigen, such as nestin. In 1990, its expression was identified in most neuronal precursor cells and the morphological development of neuronal and glial cells. Regarding the TuJ-1 marker, it is part of the beta-tubulin family. Its role in mitosis and motility in neuronal cells has been reported with identification in processes such as neurogenesis, development, and axon maintenance. Menezes and Luskin [24] used it for labeling post-mitotic immature neuronal cells. Identification of the GFAP antigen or marker together with nestin has been reported in proliferation states of neuronal precursor cells and alone in stages of glial cell differentiation, particularly astrocytes [3, 25, 26].

With the positive results obtained in our research with immunocytochemistry of the neural markers nestin, TuJ-1, and GFAP, we can state that dental pulp cells from deciduous teeth, treated with neuronal induction, behave like neuronal precursor cells with the ability to mature to neuronal and glial lineages. We can also consider their pluripotent behavior and the ability to originate mesoderm and ectoderm-derived cell lineages.

Regarding the ethical and therapeutic aspects of stem cells obtained from human embryo tissue and induced pluripotent stem cells, the ethical considerations of dental pulp from deciduous teeth are not controversial because the tissue sample obtained from dental pulp is considered biological waste that causes no harm to life. On the other hand, although induced pluripotent cells have been regarded as superior to those obtained from embryo tissue, genomic instability that can form cell tumors in differentiation assays has been identified. This finding has been associated with the reprogramming process; therefore, this cell line is not considered safe for clinical application. Thus, we can consider SHEDs a potential source of cells for inclusion in personalized regenerative medicine therapies [27, 28].

Our line of research began in the year 2000, identifying mesenchymal stem cells in human dental pulp [20] that were subsequently identified in primary dentition [21, 29]. Their ability to differentiate when induced to form bone, cartilage, muscle, and even dentine, has been identified in several studies [21, 30]. Their ability to differentiate to neuronal lineage, as in the case of neural precursor cells, is one of the newest fields in cell research worldwide. Particularly in Mexico, this line of research in dentistry and its relationship with the neurosciences is being consolidated.

## Conclusions

Our findings on neurodifferentiation show that SSEA-4 positive SHEDs have a behavior similar to neuronal precursor cells. Therefore, we can suggest that the human dental pulp of primary teeth is a promising source of MSCs that could be used in regeneration therapies related to neurodegenerative diseases and peripheral nerve disorders.

## Data Availability

Data are available from the corresponding author upon reasonable request.
